# Editorial for Special Issue “Antibiotic Combination Therapy: A Strategy to Overcome Bacterial Resistance”

**DOI:** 10.3390/biomedicines13010129

**Published:** 2025-01-08

**Authors:** Helal F. Hetta, Yasmin N. Ramadan, Israa M. S. Al-Kadmy

**Affiliations:** 1 Division of Microbiology, Immunology and Biotechnology, Department of Natural Products and Alternative Medicine, Faculty of Pharmacy, University of Tabuk, Tabuk 71491, Saudi Arabia; helalhetta@aun.edu.eg; 2 Department of Medical Microbiology and Immunology, Faculty of Medicine, Assiut University, Assiut 71515, Egypt; 3 Department of Microbiology and Immunology, Faculty of Pharmacy, Assiut University, Assiut 71515, Egypt; yasmine_mohamed@pharm.aun.edu.eg; 4 Branch of Biotechnology, Department of Biology, College of Science, Mustansiriyah University, Baghdad P.O. Box 10244, Iraq

Multidrug-resistant (MDR) bacterial infections have emerged as a critical global health threat, challenging the efficacy of existing antibiotics and undermining advances in modern medicine [1,2,3]. The World Health Organization (WHO) has consistently highlighted antimicrobial resistance (AMR) as among the top ten global public health threats [4,5]. MDR pathogens, commonly referred to as “superbugs”, jeopardize the treatment of common infections and compromise the success of existing complex medical procedures, such as organ transplants, chemotherapy, and intensive care treatments [6,7]. This crisis is further exacerbated by the limited development of new antibiotics and the rapid evolution of antibiotic resistance mechanisms.

In response to this mounting challenge, this Special Issue of *Biomedicines* provides a platform for innovative research and critical reviews that aim to expand our understanding of MDR pathogens and explore promising solutions. This collection of articles spans a multitude of topics, from the potential of bacteriophage therapy to plant-based antimicrobials and the molecular mechanisms of resistance, reflecting the breadth and urgency of efforts required to combat AMR. By integrating empirical findings and theoretical insights, this Special Issue underscores the need for interdisciplinary collaboration to address this pressing public health concern.

The Special Issue features one original research article and four review articles, each addressing critical aspects of MDR bacterial infections, as outlined below.

Murtaza et al. [8] demonstrated that sodium alginate-based magnesium oxide (MgO) nanoparticles, coupled with antibiotics, effectively combat *Staphylococcus aureus* infections in Houbara bustard birds. Their study combines nanotechnology with conventional antibiotics (such as tylosin, ampicillin, and cefoxitin) to enhance antibacterial activity, showing that the nanoparticles improve the effectiveness of antibiotics while maintaining biocompatibility and reducing cytotoxicity. This approach offers a promising solution to combat antimicrobial resistance by overcoming the common limitations of traditional antibiotics, such as their low bioavailability and the resistance development associated with them. The findings of this study highlight the potential of nanoparticle-based therapies in both veterinary and human medicine.

Hetta et al. [9] reviewed the potential of phage therapy as a promising treatment for infective endocarditis caused by MDR bacteria. They highlighted the growing threat of antibiotic-resistant pathogens in the treatment of endocarditis, a serious condition that can be life-threatening if left untreated. This review emphasized the advantages of bacteriophage therapy, particularly its specificity in targeting bacteria without harming host cells. Hetta et al. [9] concluded that phage therapy represents a viable alternative or adjunct to conventional antibiotics, especially for MDR infections, and recommended further clinical trials to validate its effectiveness in treating endocarditis.

Husna et al. [10] reviewed the global challenge posed by ESBL-producing bacteria, which are resistant to a wide range of antibiotics, particularly β-lactams. They discussed the mechanisms of ESBL production and highlighted pathogens such as *Escherichia coli* and *Klebsiella pneumoniae*, which are commonly involved in ESBL-related infections. The authors provided examples of current diagnostic methods, such as the double-disk diffusion method and molecular testing, which help identify ESBL-producing organisms. In terms of treatment, Husna et al. [10] emphasized alternative effective therapeutic strategies such as carbapenem-sparing strategies that include the administration of non-carbapenem β-lactams (ceftolozane–tazobactam, ceftazidime–avibactam, temocillin, cephamycins, and cefepime) and non-β-lactams (aminoglycosides, quinolones, tigecycline, eravacycline, and fosfomycin).

Moiketsi et al. [11] explored the antimicrobial potential of various African medicinal plants as alternatives to conventional antibiotics in combating multidrug-resistant bacteria. They cited examples such as *Combretum micranthum*, *Terminalia catappa*, *Artemisia afra*, *Vanguera infausta*, and *Adansonia digitata*, which have demonstrated activity against resistant pathogens like *S. aureus*, *P. aeruginosa*, and *E. coli*. This review highlighted the promising bioactive compounds, such as tannins, flavonoids, and alkaloids, present in these plants, which contribute to their antimicrobial effects. Moiketsi et al. [11] also emphasized the importance of identifying the specific mechanisms through which these plants exert their antimicrobial activity and suggested that further research could lead to the development of plant-based therapeutics to complement or replace traditional antibiotics.

Verdial et al. [12] examined the mechanisms behind antibiotic and biocide resistance in *P. aeruginosa*, a critical pathogen in hospital-acquired infections. The review emphasized the role of biofilms, which contribute to the persistence of *P. aeruginosa* in hospital environments, making it difficult to eradicate using standard disinfectants or antibiotics. The authors highlighted the increasing concern of biocide tolerance, which can be linked to co- and cross-resistance to antibiotics. This tolerance is often a result of continuous exposure to subinhibitory concentrations of biocides, commonly encountered in healthcare, industrial, and domestic settings. *P. aeruginosa*’s ability to adapt to both biocides and antibiotics complicates infection control efforts. Tiwari et al. concluded that it is an urgent necessity to continue investing in scientific research to better understand *P. aeruginosa*’s resistance mechanisms and, consequently, to develop new control strategies to combat this pathogen. These strategies will be crucial to impeding its dissemination in hospital environments and managing the growing threat of resistance in clinical settings.

## References

[1] Masadeh M.M., Bany-Ali N.M., Khanfar M.S., Alzoubi K.H., Masadeh M.M., Al Momany E.M. (2025). Synergistic Antibacterial Effect of ZnO Nanoparticles and Antibiotics against Multidrug-resistant Biofilm Bacteria. Curr. Drug Deliv..

[2] Abdel-Raheem S.M., Abouelhassan E.M., Mandour M., El-Ghareeb W.R., Shawky M., Eltarabili R.M. (2025). Novel natural and economic approach for controlling methicillin-resistant Staphylococcus aureus using apple cider vinegar. Microb. Pathog..

[3] Pacios O., Blasco L., Bleriot I., Fernandez-Garcia L., Bardanca M.G., Ambroa A., López M., Bou G., Tomás M. (2020). Strategies to Combat Multidrug-Resistant and Persistent Infectious Diseases. Antibiotics.

[4] World Health Organization (2022). Global Antimicrobial Resistance and Use Surveillance System (GLASS) Report 2022.

[5] World Health Organization (2019). Ten Threats to Global Health in 2019. https://www.who.int/news-room/spotlight/ten-threats-to-global-health-in-2019.

[6] Algammal A., Hetta H.F., Mabrok M., Behzadi P. (2023). Editorial: Emerging multidrug-resistant bacterial pathogens “superbugs”: A rising public health threat. Front. Microbiol..

[7] Salam A., Al-Amin Y., Salam M.T., Pawar J.S., Akhter N., Rabaan A.A., Alqumber M.A.A. (2023). Antimicrobial Resistance: A Growing Serious Threat for Global Public Health. Healthcare.

[8] Murtaza M., Aqib A.I., Khan S.R., Muneer A., Ali M.M., Waseem A., Zaheer T., Al-Keridis L.A., Alshammari N., Saeed M. (2023). Sodium Alginate-Based MgO Nanoparticles Coupled Antibiotics as Safe and Effective Antimicrobial Candidates against Staphylococcus aureus of Houbara Bustard Birds. Biomedicines.

[9] Hetta H.F., Rashed Z.I., Ramadan Y.N., Al-Kadmy I.M.S., Kassem S.M., Ata H.S., Nageeb W.M. (2023). Phage Therapy, a Salvage Treatment for Multidrug-Resistant Bacteria Causing Infective Endocarditis. Biomedicines.

[10] Husna A., Rahman M., Badruzzaman A.T.M., Sikder M.H., Islam M.R., Rahman T., Alam J., Ashour H.M. (2023). Extended-Spectrum β-Lactamases (ESBL): Challenges and Opportunities. Biomedicines.

[11] Moiketsi B.N., Makale K.P.P., Rantong G., Rahube T.O., Makhzoum A. (2023). Potential of Selected African Medicinal Plants as Alternative Therapeutics against Multi-Drug-Resistant Bacteria. Biomedicines.

[12] Wajid Odhafa M., Al-Kadmy I., Pourmand M.R., Naderi G., Asadian M., Ghourchian S., Douraghi M. (2024). The context of *bla*_OXA−23_ gene in Iraqi carbapenem-resistant *Acinetobacter baumannii* isolates belonging to global clone 1 and global clone 2. BMC Res. Notes.

